# A preliminary study of ECT2 as a novel biomarker for assessing liver fibrosis and prognosis in biliary atresia

**DOI:** 10.3389/fped.2026.1879864

**Published:** 2026-06-29

**Authors:** Yuqiang Chen, Xin Li, Qianhui Yang, Yu Meng, Alimujiang Abudureyimu, Jianghua Zhan

**Affiliations:** 1 Department of General Surgery, Urumqi Children's Hospital, Xinjiang, China; 2 General Surgery Department, Tianjin Children's Hospital, Tianjin, China

**Keywords:** biliary atresia, biomarker, epithelial cell transforming sequence 2, liver fibrosis, prognosis

## Abstract

**Background & aims:**

Biliary atresia (BA) is the leading cause of cholestatic liver disease and cirrhosis in infancy. Currently, there is a lack of efficient biomarkers for early diagnosis and prognostic evaluation of this disease, which urgently needs to be addressed. This study aimed to screen and validate key biomarkers closely associated with BA liver fibrosis progression and prognosis through bioinformatics analysis combined with clinical sample verification.

**Methods:**

First, the public transcriptomic dataset GSE122340 was thoroughly analyzed to screen differentially expressed genes, followed by construction of a protein–protein interaction (PPI) network. Epithelial cell transforming sequence 2 (ECT2) was ultimately identified as the core hub gene. Subsequently, clinical samples were collected, including 20 cases in the BA group (stratified by liver fibrosis severity) and 10 cases in the choledochal cyst control group. The expression level of ECT2 was clinically verified using immunohistochemistry, real-time quantitative polymerase chain reaction (qPCR), and enzyme-linked immunosorbent assay (ELISA). Receiver operating characteristic (ROC) curve analysis was performed to validate the predictive efficacy of serum ECT2 level for BA diagnosis, fibrosis stratification, and postoperative native liver survival. Kaplan–Meier (KM) curve analysis was used to verify the relationship between serum ECT2 level and postoperative native liver survival.

**Results:**

ECT2 was significantly highly expressed in liver tissue (both mRNA and protein levels) and serum of BA patients, and its expression intensity was positively correlated with the severity of liver fibrosis. ROC curve analysis showed that serum ECT2 exhibited favorable efficacy in BA diagnosis, liver fibrosis stratification, and prediction of 2-year postoperative native liver survival, with area under the curve (AUC) values of 0.906, 0.807, and 0.844.Compared with MMP7, it demonstrates comparable predictive performance in the diagnosis of BA (AUC = 0.924) and the stratification of liver fibrosis (AUC = 0.829), and outperforms MMP7 in predicting autologous liver survival (AUC = 0.767). Its overall predictive performance is superior to that of traditional liver function markers (GGT, TBIL, TBA). Kaplan–Meier survival analysis further confirmed that BA patients with high serum ECT2 expression had significantly reduced native liver survival rates postoperatively.

**Conclusion:**

This study systematically demonstrates that ECT2 is characteristically highly expressed in BA and its association with disease fibrosis severity and poor prognosis. Preliminary findings suggest that serum ECT2 as a promising non-invasive biomarker, providing new technical approaches and theoretical basis for clinical diagnosis, disease assessment, and prognostic judgment of BA.

## Introduction

Biliary atresia (BA) is a highly destructive cholestatic liver disease in infancy and the leading cause of end-stage liver disease and liver transplantation in children (1, 2). Its pathological features are characterized by progressive inflammation and fibrotic obliteration of intrahepatic and extrahepatic bile ducts, leading to impaired bile drainage, intrahepatic cholestasis, and eventually secondary liver fibrosis and cirrhosis (3). Kasai portoenterostomy (KPE) is the first-line surgical procedure for BA, with the core goal of restoring bile drainage. However, the native liver survival rate of children after surgery is unsatisfactory, and most children ultimately require liver transplantation to save their lives (4). Therefore, in-depth exploration of the pathogenesis of BA and screening of reliable biomarkers for early diagnosis, accurate assessment of disease progression, and prognosis prediction are of great practical significance for optimizing the clinical management model and improving treatment decisions for children.

Currently, there are still many shortcomings in the clinical diagnosis and disease assessment of BA: the diagnostic process primarily relies on imaging studies and invasive procedures, with a lack of specific serological markers to support it; postoperative prognosis is often evaluated using traditional liver function indicators such as total bilirubin and γ-glutamyltransferase, which are easily influenced by various factors and have poor specificity (5, 6). Transcriptomics studies have identified abnormal gene expression in multiple pathways—including immune inflammation, extracellular matrix remodeling, and lipid metabolism—within BA liver tissue. However, most studies remain at the descriptive analysis stage, lacking a comprehensive research framework that spans from bioinformatics screening to multi-level validation in independent clinical cohorts. Consequently, most differentially expressed genes cannot be translated into diagnostic or prognostic markers with practical clinical value (7).

Epithelial Cell Transforming Sequence 2 (ECT2) belongs to the guanine nucleotide exchange factor family and plays a key role in regulating the cell cycle, cell division, and tumorigenesis (8). However, the expression patterns, specific functions, and clinical significance of ECT2 in non-neoplastic liver diseases remain unclear, and research on its role in biliary atresia is particularly lacking. Preliminary analysis in this study revealed that ECT2 occupies a central regulatory position within the BA differential gene network, suggesting that it may be involved in the pathophysiological processes of BA and play a significant role.

This study aims to identify key hub genes in BA using public datasets, validate the expression patterns of the candidate gene ECT2 at the tissue and serum levels using clinical specimens, and evaluate its clinical potential as a non-invasive prognostic biomarker for BA.

## Methods

### Data collection, preprocessing, and screening of differentially expressed genes

The biliary atresia dataset GSE122340 used in this study was downloaded from the GEO database (9). The raw data were processed using R (version 4.2.1). After data cleaning, standardization was performed using the “preprocessCore” package (version 1.58.0) (10) and quantile normalization.

Subsequent differential expression analysis was performed using the limma package (version 3.54.2) (3). The criteria for identifying differentially expressed genes were as follows: *p*.adjust < 0.05 (Benjamini–Hochberg method) and absolute log2 fold change (|log2FC|) > 0.58. Volcano plots were generated using the ggplot2 package [3.4.4] to display the distribution of all genes.

### Functional enrichment analysis of differentially expressed genes

Enrichment analysis of Gene Ontology (GO) and Kyoto Encyclopedia of Genes and Genomes (KEGG) pathways was performed on the differentially expressed genes. After converting the IDs of the input molecular list based on the human database org.Hs.eg.db, enrichment analysis was performed using the clusterProfiler [4.4.4] package (11). The z-score for each enriched term was calculated using the logFC values of the provided molecules via the GOplot [1.0.2] package (12). A *p*-adjusted value of <0.05 was used as the criterion for functional enrichment.

We performed further GSEA analysis using the clusterProfiler package (13), employing the c2.cp.all.v2022.1.Hs.symbols.gmt gene set from the MSigDB database as the annotation library. We set the significance thresholds at FDR (q-value) < 0.25 and *p*.adjust < 0.05 to identify statistically significant enriched pathways.

### PPI network construction and core gene selection

Import the list of differentially expressed genes obtained in the previous step into the STRING database to construct a PPI network, setting the organism to “Human,” the confidence threshold to medium (0.400), and hiding disconnected nodes in the network. Export the resulting interaction data in TSV format to Cytoscape (version 3.10.4). Use the MCODE plugin to identify highly interconnected functional modules within the network. Subsequently, employ various algorithms from the cytoHubba plugin to rank the genes within the highest-scoring modules by importance, thereby identifying core hub genes in the network.

### Patient selection and tissue sampling

This study conducted a retrospective analysis of clinical data from children with biliary atresia (BA) who underwent surgical treatment in the Department of General Surgery at Tianjin Children's Hospital between December 2021 and December 2024. Clinical data collected included gender, age at surgery, preoperative laboratory tests, and autologous liver survival at 2 years postoperatively. Case inclusion followed the following criteria: (1) Diagnosis of type III BA confirmed by intraoperative cholangiography combined with postoperative pathological examination. (2) A liver tissue specimen from the anterior margin of the right hepatic lobe was obtained during surgery. Exclusion criteria: (1) Incomplete case records; (2) Concurrent congenital diseases, infectious diseases, or systemic malformations. A total of 20 children with BA were included in the BA group. BA-specific liver fibrosis was classified into four grades based on the grading system proposed by Xu et al. (14). Children in the BA group were further divided into two subgroups based on the severity of fibrosis: the mild group (fibrosis grades I–II, *n* = 10) and the severe group (fibrosis grades III–IV, *n* = 10). Additionally, 10 pediatric patients who underwent surgery for choledochal cysts (CC) at our hospital during the same period and from whom liver tissue samples were obtained from the anterior margin of the right hepatic lobe were included as controls. All tissue samples were immediately placed in a −80 °C ultra-low temperature freezer for long-term storage after collection.

### Immunohistochemical staining

After paraffin sections underwent dewaxing and antigen retrieval, they were blocked with goat serum for 60 min. Following washing, the sections were incubated with the primary antibody (rabbit anti-human ECT2 polyclonal antibody, Bioss, China, diluted 1:200) and incubated overnight at 4 °C. After washing and gently centrifuging the sections, the corresponding secondary antibody was added, and the sections were incubated at room temperature in the dark for 60 min. Subsequently, 3,3′-diaminobenzidine tetrahydrochloride (DAB) solution was added for color development. The development process was monitored under a microscope, and after completion, the sections were counterstained with hematoxylin.

The sections were examined using an optical microscope, and for each section, images were captured from five randomly selected, non-overlapping fields of view at ×100 magnification. Semi-quantitative analysis was performed using ImageJ software, with the average optical density (OD) value serving as the evaluation metric. This value was calculated as the ratio of the total optical density of positive cells within a single field of view to the total number of valid pixels.

### Real-time quantitative polymerase chain reaction

Following the instructions for the Superbrilliant® TRI RNA Lysis Kit (Tianjin Zhongshi Gene Technology Co., Ltd.), total RNA was extracted from frozen liver tissue and cells, and first-strand cDNA was synthesized via reverse transcription. RT-qPCR was then performed using the Superbrilliant® 3rd Generation ZAPA SYBR Green qPCR Master Mix (2×) Kit (Tianjin Zhongshi Gene Technology Co., Ltd.). Reaction protocol: 300 s pre-denaturation at 95 °C, followed by 40 amplification cycles (95 °C for 10 s, 60 °C for 20 s). Glyceraldehyde-3-phosphate dehydrogenase (GAPDH) was used as the internal control, and relative mRNA expression levels were calculated using the 2^(-ΔΔCT) method. The primer sequences used were: GAPDH-F: 5′-GGA GG, TCC CTC CAA AAT-3′; GAPDH-R: 5′-GGC TGT CAT ACT TCT CAT GG-3′; ECT2-F: 5′-ACT GGG AGG ACT AGC TTG-3′, ECT2-R: 5′-CAC TCT TGT TTC AAT CTG AGG CA-3′.

### Detection of serum ECT2 concentration by ELISA kit

3 mL of blood was collected from children in the CC and BA groups using anticoagulant tubes. After standing at room temperature for 30 min, the samples were centrifuged at 3,000 rpm for 10–15 min within 2 h. Hemolyzed samples were discarded, and the serum was separated, aliquoted, and stored at −80 °C. An ELISA kit (catalog number MB-01151A) was used to measure ECT2 concentrations. ELISA Kit (Catalog No. m1950502 V) for measuring MMP7 levels. A total of 30 serum samples and 5 concentration gradient standards (all with duplicate wells) were tested. The procedure was strictly followed according to the instructions. OD values were measured at 450 nm using a microplate reader. Samples exceeding the calibration range were diluted 1:5 and retested. A linear equation was fitted using the standard concentrations and OD values to calculate the serum ECT2 concentration.

### Statistical analysis

Statistical analyses were performed using R version 4.2.1. For the Shapiro–Wilk normality test and Levene's test of homogeneity of variances for the measurement data. Continuous variables that followed a normal distribution are expressed as x¯ ± s, and comparisons between groups were performed using an independent samples *t*-test; If the data are normally distributed but have unequal variances, use a corrected *t*-test; continuous variables that did not follow a normal distribution are expressed as the median (interquartile range) M(Q1, Q3), and comparisons between groups were performed using the Mann–Whitney *U* test (Wilcoxon rank-sum test); comparisons among multiple groups were performed using one-way ANOVA, when variances were homogeneous, Tukey's HSD test was used for *post-hoc* comparisons; when variances were heterogeneous, the nonparametric Kruskal–Wallis test was used. Correlations between two continuous variables with a normal distribution were analyzed using Pearson's correlation coefficient, while variables with a non-normal distribution were analyzed using Spearman's rank correlation coefficient. Autologous liver survival curves were plotted using the Kaplan–Meier method. Differences in survival rates between groups were calculated using the Log-rank proportional hazards model. All statistical tests were two-sided, and a *P*-value < 0.05 was considered statistically significant.

## Results

### Screening and functional enrichment analysis of differentially expressed genes in biliary atresia

**Figure 1:**
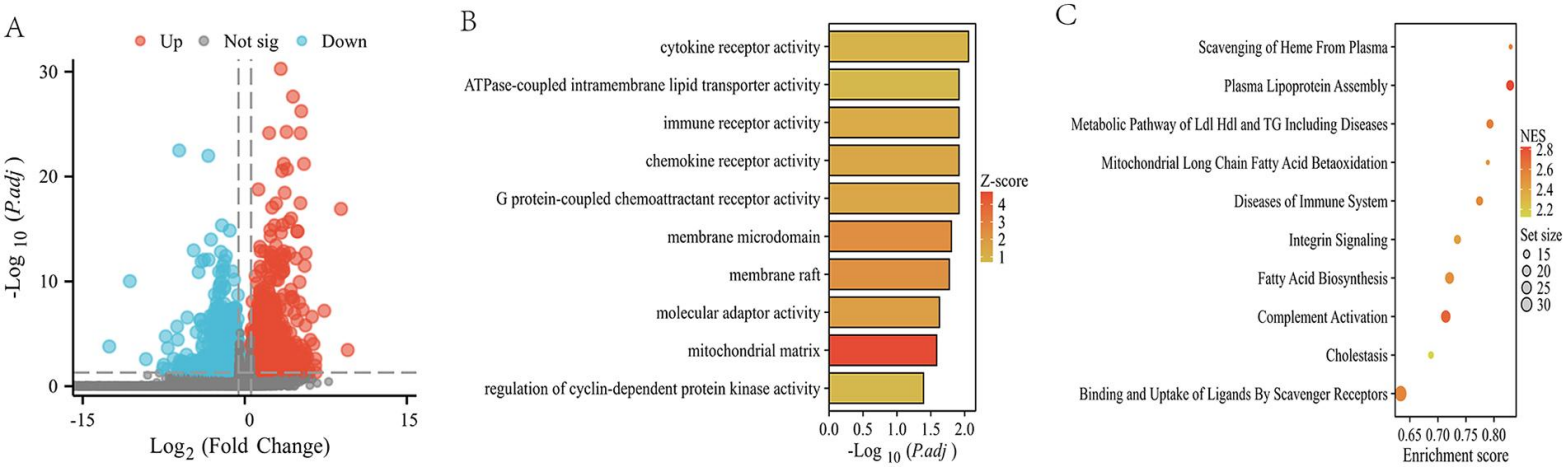
Screening and functional enrichment analysis of differentially expressed genes in biliary atresia. **(A)** Volcano plot of differentially expressed genes in GSE122340. **(B)** Bar chart of GOKEGG enrichment analysis in GSE122340. Z-score, normalized enrichment score. **(C)** Bubble plot of GSEA enrichment analysis in GSE122340. NES, normalized enrichment score.

The GSE122340 dataset contains RNA sequences from liver biopsies of 171 children with biliary atresia and 7 healthy controls. Gene differential expression analysis was performed on the dataset according to predefined criteria, and a volcano plot was generated. The results showed that 868 genes were upregulated and 720 genes were downregulated in the GSE122340 dataset (Figure 1A); GOKEGG gene functional enrichment analysis revealed that differentially expressed genes were primarily enriched in pathways related to immune receptors, chemokine receptor signaling, membrane function, and lipid transport, accompanied by upregulation of mitochondrial matrix function and alterations in cell cycle kinase regulation (Figure 1B). GSEA analysis results showed that differentially expressed genes were significantly enriched in pathways related to lipid metabolism, immune inflammation, and hepatobiliary function (Figure 1C).

### Constructing a PPI network and identifying key genes

**Figure 2:**
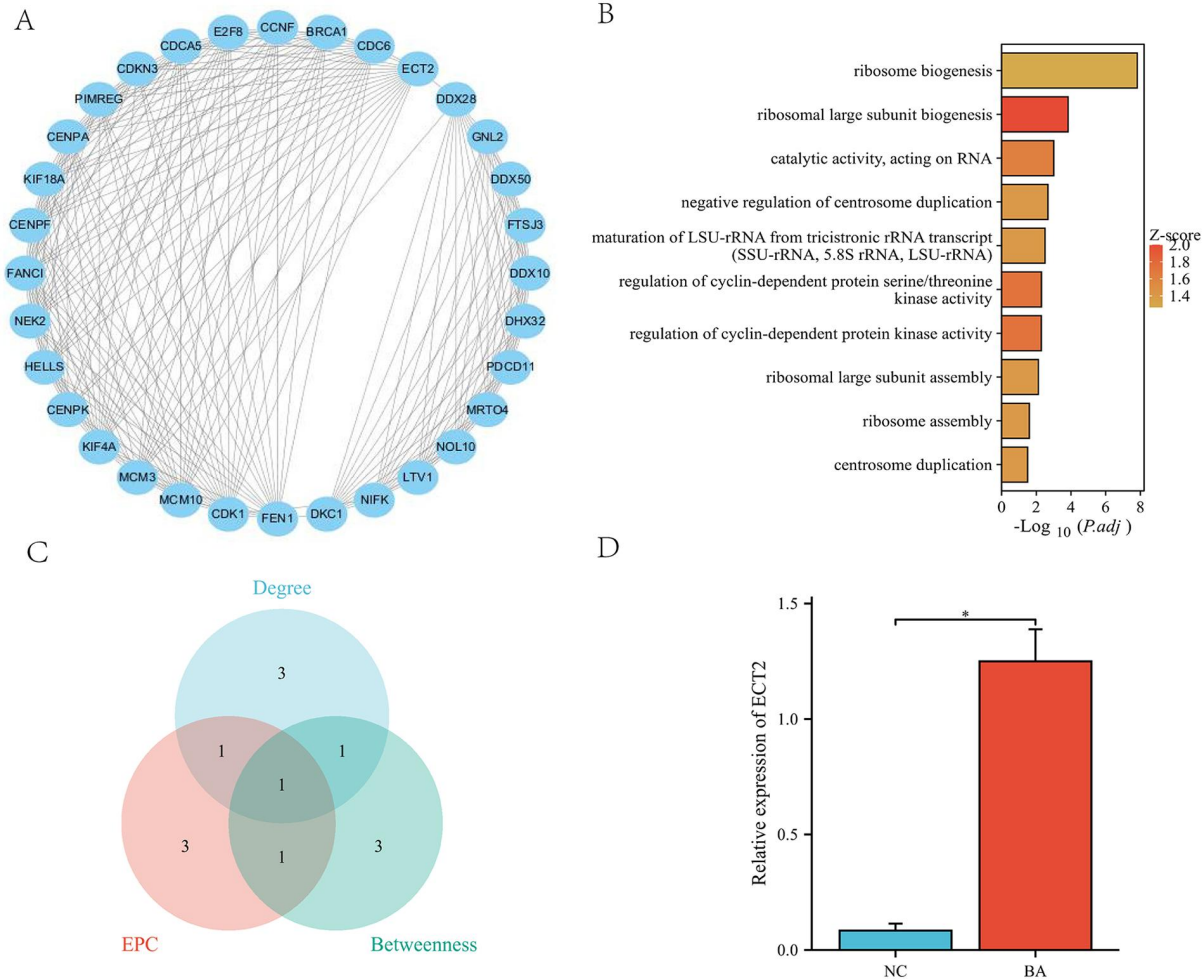
Constructing a PPI network and identifying key genes. **(A)** The MCODE plugin identifies the most highly interconnected modules in the PPI network. **(B)** GOKEGG analysis of genes in the most highly interconnected modules. **(C)** The cytoHubba plugin screens for core genes. **(D)** Expression analysis of ECT2 in the GSE122340 dataset.

The list of differentially expressed genes was imported into the STRING database to construct a PPI network. The resulting interaction data in TSV format was exported to Cytoscape software, and the MCODE plugin was used to identify highly interconnected functional modules within the network; the module with the highest score contained 32 genes (Figure 2A). GOKEGG enrichment analysis was performed on these 32 factors, and the results showed that they were significantly enriched in pathways related to ribosome biosynthesis and cell cycle regulation (Figure 2B). Subsequently, various algorithms in the cytoHubba plugin were used to rank the importance of these 32 genes and identify core hub genes in the network. By intersecting the top six factors from the Degree, EPC, and Betweenness3 methods, ECT2 was identified as the sole core gene (Figure 2C). Expression analysis of the GSE122340 dataset showed that ECT2 expression levels in liver tissue samples from patients with biliary atresia were significantly higher than those in normal control samples (U = 323, *P* = 0.0396) (Figure 2D).

### Table of baseline characteristics of pediatric patients and multivariate logistic regression analysis of ECT2

Significant disparities in multiple baseline characteristics were observed between the CC and BA groups (Table 1), as well as between mild and severe BA subgroups (Table 2); such discrepancies may act as confounding variables and interfere with the diagnostic and prognostic predictive performance of ECT2. To compare the predictive performance of ECT2 and MMP7, serum ECT2, serum MMP7, and baseline variables with significant intergroup differences were included in a univariate logistic regression model (Table 3). Due to sample size limitations and the EPV rule (number of events/number of independent variables ≥ 10), only two variables could be included in the multivariate regression analysis. Based on the *P*-values from the univariate analysis and the relevance to the study, TBIL and ECT2 were included in the multivariate analysis; the results showed that ECT2 was an independent predictor of BA (OR = 1.005, 95% CI: 1.001–1.009, *P* = 0.027).

**Table 1:** Baseline characteristics of the CC and BA groups.

Characteristics	CC	BA	*P* value
*n*	10	20	
Sex, *n* (%)			1.000
Male	5 (16.7%)	10 (33.3%)	
Female	5 (16.7%)	10 (33.3%)	
Age at surgery (days)	134.5 ± 65.629	62.7 ± 23.407	0.007
Grading of BA-specific fibrosis, *n* (%)			1.000
I	0 (0%)	3 (15%)	
II	0 (0%)	7 (35%)	
III	0 (0%)	4 (20%)	
IV	-	6 (30%)	
ALB (g/L)	68.95 (62.525, 73.05)	41.55 (37.85, 43.8)	<0.001
ALT (U/L)	31.5 (20.5, 97)	106 (65.25, 146.75)	0.048
AST (U/L)	41 (32.75, 123.5)	168.5 (124.25, 250.5)	0.013
ALP (U/L)	422.4 ± 231.58	603.25 ± 236.84	0.057
LDH (U/L)	319.5 (267, 359.5)	349 (287.75, 498.5)	0.244
GGT (U/L)	76.5 (26.25, 186.25)	355 (223, 542.25)	0.002
CHE (U/L)	7,596.2 ± 1,993.3	5,595.4 ± 1,476.7	0.006
TBIL (μmol/L)	42.65 (8.65, 122.35)	156.6 (135.15, 200.9)	0.005
DBIL (μmol/L)	33.85 (3.95, 93.575)	122.25 (102.75, 156.32)	0.005
IBIL (μmol/L)	16.47 ± 15.664	36.69 ± 16.893	0.004
TBA (μmol/L)	46 (5.25, 124.5)	107.5 (96.5, 122.5)	0.166
CRP (mg/L)	2.1 (2, 2.175)	2.45 (2.275, 2.85)	0.003
WBC (×10^9^/L)	8.335 (6.545, 8.97)	10.985 (9.455, 14.5)	0.011
RBC (×10^12^/L)	4.144 ± 0.67034	3.3805 ± 0.48696	0.001
HGB (g/L)	116.9 ± 15.423	106.4 ± 17.203	0.115
PLT (×10^9^/L)	363.7 ± 117.05	442.8 ± 168.15	0.194

**Table 2:** Baseline characteristics of the BA mild and BA severe groups.

Characteristics	BA mild	BA severe	*P* value
*n*	10	10	
Sex, *n* (%)			0.656
0	6 (30%)	4 (20%)	
1	4 (20%)	6 (30%)	
Age at surgery (days)	57.4 ± 25.816	68 ± 20.683	0.324
Grading of BA-specific fibrosis, *n* (%)			<0.001
I	3 (15%)	0 (0%)	
II	7 (35%)	0 (0%)	
III	0 (0%)	4 (20%)	
IV	0 (0%)	6 (30%)	
ALB (g/L)	41.55 (37.475, 43.425)	41.35 (38.35, 44.05)	0.684
ALT (U/L)	88.7 ± 35.059	161.5 ± 108.2	0.068
AST (U/L)	169 (125.25, 245.5)	168.5 (127.75, 256.25)	0.821
ALP (U/L)	641.9 ± 308.12	564.6 ± 141.99	0.484
LDH (U/L)	304 (277, 389.75)	448 (297.75, 513.5)	0.307
GGT (U/L)	426 (168, 584.75)	310.5 (252, 431.5)	0.796
CHE (U/L)	5,967 ± 1,651.3	5,182.6 ± 1,214.2	0.259
TBIL (μmol/L)	176.97 ± 69.856	165.32 ± 42.462	0.658
DBIL (μmol/L)	115.65 (104.63, 157.38)	129.25 (106.03, 152.4)	1.000
IBIL (μmol/L)	38.55 ± 22.195	34.83 ± 10.108	0.638
TBA (μmol/L)	116 ± 38.442	111 ± 23.319	0.729
CRP (mg/L)	2.5 (2.325, 3.55)	2.35 (2.225, 2.5)	0.444
WBC (×10^9^/L)	11.989 ± 5.1218	12.366 ± 3.7945	0.854
RBC (×10^12^/L)	3.428 ± 0.53849	3.333 ± 0.45346	0.675
HGB (g/L)	110.7 ± 18.856	102.1 ± 15.103	0.275
PLT (×10^9^/L)	411.9 ± 163.83	473.7 ± 175.29	0.426

Severity is based on histology rather than laboratory values.

**Table 3:** Univariate and multivariate logistic regression analysis.

Characteristics	Univariate analysis		Multivariate analysis	
	Odds ratio (95% CI)	*P* value	Odds ratio (95% CI)	*P* value
Age at surgery (days)	0.957 (0.925–0.991)	0.012		
ALB (g/L)	0.861 (0.784–0.944)	0.002		
ALT (U/L)	1.003 (0.994–1.011)	0.510		
AST (U/L)	1.008 (0.999–1.016)	0.085		
GGT (U/L)	1.006 (1.000–1.011)	0.039		
CHE (U/L)	0.999 (0.999–1.000)	0.018		
TBIL (μmol/L)	1.021 (1.005–1.038)	0.011	1.020 (1.000–1.040)	0.046
DBIL (μmol/L)	1.025 (1.005–1.044)	0.013		
IBIL (μmol/L)	1.099 (1.021–1.184)	0.012		
TBA (μmol/L)	1.000 (0.992–1.009)	0.920		
CRP (mg/L)	65.702 (1.062–4,063.472)	0.047		
WBC (×10^9^/L)	1.392 (1.010–1.918)	0.043		
RBC (×10^12^/L)	0.084 (0.013–0.545)	0.009		
ECT2 (pg/mL)	1.004 (1.001–1.007)	0.006	1.005 (1.001–1.009)	0.027
MMP7 (ng/mL)	1.328 (0.870–2.026)	0.189		

To further verify the predictive capacity of ECT2 for postoperative native liver survival, we conducted univariate and multivariate Cox regression analyses (Table 4). Based on the *P*-values from the univariate analysis, AST and ECT2 were included in the multivariate analysis. The results showed that ECT2 can predict autologous liver survival in pediatric patients to a certain extent (OR = 1.002, 95% CI: 1.001–1.004, *P* = 0.025).

**Table 4:** Univariate and multivariate Cox analyses.

Characteristics	Univariate analysis		Multivariate analysis	
	Odds ratio (95% CI)	*P* value	Odds ratio (95% CI)	*P* value
Age at surgery (days)	1.015 (0.991–1.040)	0.232		
ALB (g/L)	0.961 (0.881–1.049)	0.373		
ALT (U/L)	1.004 (0.997–1.011)	0.275		
AST (U/L)	1.010 (1.004–1.016)	0.002	1.007 (1.001–1.013)	0.034
GGT (U/L)	1.001 (0.999–1.002)	0.277		
CHE (U/L)	1.000 (1.000–1.000)	0.784		
TBIL (μmol/L)	1.002 (0.991–1.014)	0.728		
DBIL (μmol/L)	1.006 (0.993–1.019)	0.381		
IBIL (μmol/L)	0.982 (0.945–1.021)	0.368		
CRP (mg/L)	1.025 (0.921–1.142)	0.651		
WBC (×10^9^/L)	0.909 (0.784–1.053)	0.204		
RBC (×10^12^/L)	0.471 (0.152–1.459)	0.192		
ECT2 (pg/mL)	1.002 (1.001–1.004)	0.006	1.002 (1.000–1.004	0.025
MMP7 (ng/mL)	1.025 (0.998–1.053)	0.075		

### ECT2 is up-regulated in liver tissue and serum of BA patients and positively correlated with liver fibrosis severity

**Figure 3:**
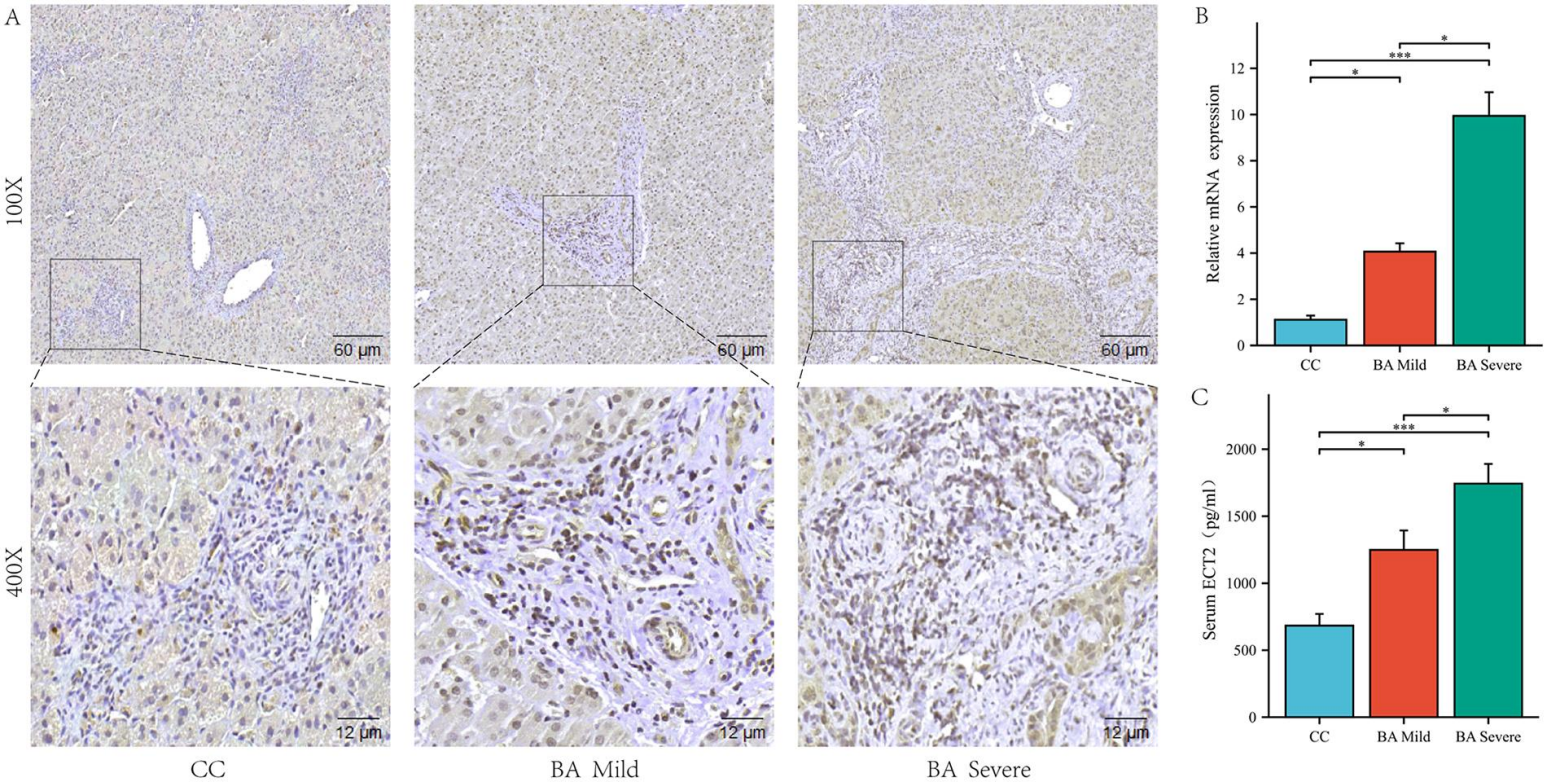
ECT2 is up-regulated in liver tissue and Serum of BA patients and positively correlated with liver fibrosis severity. **(A)** ECT2 immunohistochemical staining in each group. **(B)** Relative mRNA expression levels of ECT2 in each group. **(C)** Serum ECT2 concentrations in each group.

IHC analysis of liver tissue samples from the CC group, BA mild group, and BA severe group showed that ECT2-positive brown staining is primarily localized to the cytoplasm of hepatocytes and cells in the portal area; scattered positive staining granules are visible in the nuclei of some cells. In the CC group, the positive signal was weak, mainly distributed in the cytoplasm as fine granular diffusion, and only very few weak positive staining was seen in the nucleus; with the aggravation of BA liver fibrosis (BA Mild → BA Severe), The intensity of ECT2 staining gradually increases in hepatocytes, proliferating bile duct epithelium, and inflammatory cells infiltrating the lesion; brown cytoplasmic staining is patchily concentrated, while the extent of positive staining in the nuclear regions and the intensity of staining both show a tendency to increase (Figure 3A). Real-time qPCR analysis showed that the relative expression level of ECT2 mRNA in BA liver tissue was significantly higher than that in the CC group; in the BA group, the relative expression level of mRNA gradually increased with the aggravation of fibrosis (Figure 3B). ELISA results showed that the serum ECT2 concentration in the BA group was higher than that in the CC group, and the serum ECT2 concentration in the BA group gradually increased with the aggravation of fibrosis (Serum ECT2 concentrations: CC: 683.33 ± 276.53 pg/mL; BA Mild: 1,248.60 ± 458.19 pg/mL; BA Severe: 1,743.20 ± 465.31 pg/mL; one-way ANOVA + Tukey-HSD multiple comparisons; Overall F = 16.776, *P* < 0.001; pairwise comparisons: CC vs. BA Mild *P* = 0.0125, CC vs. BA Severe *P* < 0.001, BA Mild vs. BA Severe *P* = 0.0307 (Figure 3C).

### Value of ECT2 as a potential biomarker for diagnosis and prognostic evaluation of biliary atresia

**Figure 4:**
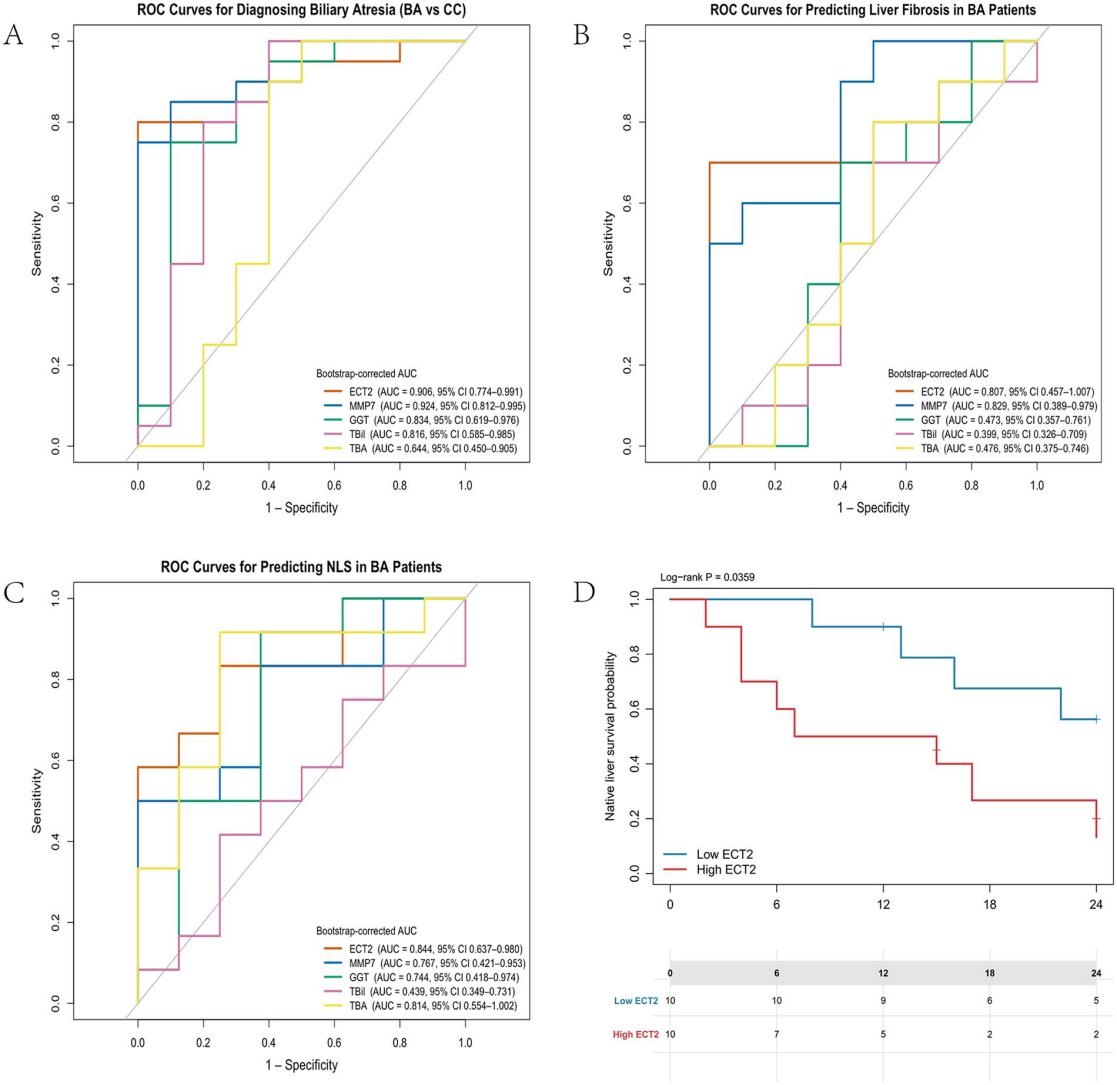
Value of ECT2 as a potential biomarker for diagnosis and prognostic evaluation of biliary atresia. **(A)** ROC curves for the diagnosis of BA using ECT2, MMP7, and conventional liver function tests. **(B)** ROC curves for predicting the severity of BA fibrosis using ECT2, MMP7, and conventional liver function tests. **(C)** ROC curves for predicting autologous liver survival after B-cell transplantation using ECT2, MMP7, and conventional liver function tests. **(D)** Kaplan–Meier survival curves for children with BA in the high- and low-expression groups of ECT2 at 2 years post-surgery.

Given the small sample size of this study (20 cases of BA and 10 cases of CC), directly calculating the AUC of the ROC curve based on a single dataset carries a risk of overfitting and an overoptimistic interpretation of the results. Therefore, for the ROC models predicting BA based on serum ECT2 levels, we performed internal validation using 2,000 bootstrap resamples to calculate the adjusted AUC and 95% confidence intervals. The results showed that, when using serum ECT2 concentrations to predict BA diagnosis, the AUC was 0.906, comparable to that of MMP7 (AUC = 0.924) and significantly higher than that of traditional liver function markers such as GGT (AUC = 0.834), TBil (AUC = 0.816), and TBA (AUC = 0.644) (Figure 4A); the AUC for predicting mild and severe liver fibrosis was 0.807, which was comparable to that of MMP7 (AUC = 0.829) and significantly higher than that of GGT (AUC = 0.473), TBil (AUC = 0.399), and TBA (AUC = 0.476) (Figure 4B); the AUC for predicting the survival of the autologous liver 2 years after BA surgery was 0.844, which was superior to that of MMP7 (AUC = 0.767) and significantly higher than that of GGT (AUC = 0.744), TBil (AUC = 0.439), and TBA (AUC = 0.814) (Figure 4C). This indicates that serum ECT2 demonstrates good predictive performance for the diagnosis of BA, fibrosis staging, and postoperative autologous liver survival. Compared with MMP7, it demonstrates comparable predictive performance for the diagnosis of BA and the staging of fibrosis, while outperforming MMP7 in predicting the survival of the native liver at 2 years post-surgery. Its overall predictive performance is significantly superior to that of traditional liver function markers. Children in the BA group were divided into high-expression and low-expression groups based on median serum ECT2 expression levels. Kaplan–Meier survival curves were plotted with 24 months post-surgery as the follow-up endpoint. Log-rank proportional hazards model analysis showed that the autologous liver survival outcome in the high-expression group was significantly worse than that in the low-expression group (*P* = 0.0359), and the 24-month autologous liver survival rate was significantly lower (Figure 4D). This suggests that high ECT2 expression is a potential predictive marker of poor autologous liver survival in children with BA.

## Discussion

Biliary atresia (BA) is a major cause of cholestatic liver disease and cirrhosis in infancy. It is characterized by progressive fibrotic and inflammatory obstruction of the intra- and extrahepatic bile ducts, ultimately leading to liver fibrosis and liver failure (15). Although previous transcriptomic studies have identified numerous differentially expressed genes in BA and established the central role of the immune system in its pathogenesis (16), there remains a lack of highly specific and sensitive biomarkers that can be effectively applied to clinical diagnosis, disease assessment, and prognosis prediction (17). Currently, traditional liver function markers commonly used in clinical practice have significant limitations in distinguishing BA from other cholestatic diseases and in assessing the degree of liver fibrosis (18); therefore, identifying superior non-invasive biomarkers is crucial for improving the clinical management of affected children. This study selected children with common bile duct cysts as the clinical control group, a common control design in current research on BA-related biomarkers. Since CC is also a congenital biliary tract surgical condition, this approach eliminates congenital biliary tract developmental abnormalities as a confounding factor; however, due to limitations in single-center case resources, complete age matching among enrolled cases could not be achieved, resulting in an imbalance in baseline age.

Using bioinformatics analysis methods, this study narrowed the focus of the complex transcriptional changes in BA to ECT2, a key hub gene. As a guanine nucleotide exchange factor, ECT2 primarily functions to activate members of the Rho GTPase family, thereby regulating the cell cycle, cytokinesis, and cytoskeletal reorganization (19). Reactive proliferation of intrahepatic bile ducts and progressive liver fibrosis are the core pathological features of BA, and these pathological processes are inseparable from abnormal cell proliferation, migration, and excessive extracellular matrix deposition (20). Based on this, high expression of ECT2 may promote the proliferation and activation of cholangiocytes and hepatic stellate cells by activating downstream signaling pathways such as Rho/ROCK, ultimately driving the processes of bile duct proliferation and fibrosis. This hypothesis is highly consistent with the findings of significant activation of cell cycle regulatory pathways identified in the functional enrichment analysis of this study, providing a reasonable biological explanation for ECT2's involvement in the pathogenesis of BA. The findings of this study extend the role of ECT2, a classic tumor-associated factor, to the field of pediatric cholestatic liver disease, offering a new research direction for a deeper understanding of the molecular regulatory mechanisms underlying BA.

IHC results from clinical tissue specimens confirmed that ECT2 expression is significantly upregulated in liver tissue from patients with BA, with staining intensity gradually increasing as the degree of liver fibrosis worsens. This suggests that ECT2 may be a key regulatory molecule in the progression of liver fibrosis, and its upregulation may be closely associated with the onset and progression of BA-related liver fibrosis. In assessing the clinical predictive value of ECT2, the results of this study showed that serum ECT2 demonstrated excellent performance in the diagnosis of BA, liver fibrosis staging, and prognosis prediction (AUCs of 0.906, 0.807, and 0.844, respectively), with overall predictive performance significantly superior to traditional liver function markers (GGT, TBIL, TBA). Compared with MMP-7, the predictive performance for BA diagnosis and fibrosis staging was comparable (AUCs of 0.924 and 0.829, respectively); although some reports have indicated that serum MMP-7 is the only significant predictor 6 weeks after KPE (21), our study showed that serum ECT2 outperformed MMP-7 in predicting autologous liver survival at 2 years post-surgery (AUC = 0.767). Furthermore, Kaplan–Meier survival analysis revealed that the autologous liver survival outcomes in the high-expression group were significantly worse than those in the low-expression group (*P* = 0.0359), further demonstrating ECT2's ability to accurately predict postoperative autologous liver survival.

This study has certain limitations. First, the clinical validation cohort had a relatively small sample size and employed a single-center retrospective study design, lack of external validation, which may have compromised statistical power; therefore, caution is warranted when generalizing the study's conclusions to a broader population. Second, this study primarily focuses on analyzing the correlation between ECT2 expression patterns and the clinical phenotype of BA; no gain-of-function or loss-of-function experiments have been conducted in cellular or animal models, so the specific molecular mechanisms by which ECT2 contributes to the progression of biliary cirrhosis remain unclear. Third, the control group consisted solely of children with common bile duct cysts. There are significant differences in the pathogenesis and pathological characteristics of cholestatic disease between the CC and BA groups. Furthermore, the age at surgery was significantly higher in the CC group than in the BA group, and some children in the CC group did not exhibit obvious jaundice. Imbalances in baseline age and the severity of cholestasis may confound and interfere with the comparison of ECT2 expression between the groups; additionally, the study did not include clinically important differential diagnoses of cholestasis, such as progressive familial intrahepatic cholestasis, Alagille syndrome, and neonatal hepatitis. The lack of serum and tissue ECT2 testing data for these conditions prevents the demonstration of ECT2's diagnostic specificity for BA across the full spectrum of infantile cholestasis diseases, which is a shortcoming of the study's control design. Future prospective trials should include cohorts of various cholestatic diseases to improve the control system.

## Conclusion

Through a systematic study, this research confirms that ECT2 can serve as a potential new biomarker for predicting the progression of liver fibrosis in patients with biliary atresia and postoperative adverse outcomes. Through a comprehensive research framework ranging from bioinformatics-based prediction to multi-level clinical validation, the study demonstrated that serum ECT2 outperforms traditional markers in the diagnosis of BA, stratification of liver fibrosis, and prognosis prediction, providing a solid foundation for its clinical translation. Future research should conduct multicenter, large-scale prospective cohort studies. Create an independent validation set to further validate the clinical utility of ECT2. Concurrently, gene editing technologies and animal models of the disease should be utilized to elucidate the functional role of ECT2 in the pathogenesis of BA, thereby laying the foundation for the development of targeted intervention strategies for BA.

## Data Availability

The raw data supporting the conclusions of this article will be made available by the authors, without undue reservation.
